# Endodontic length measurements using cone beam computed tomography with dedicated or conventional software at different voxel sizes

**DOI:** 10.1038/s41598-021-88980-4

**Published:** 2021-05-03

**Authors:** Khoa Van Pham

**Affiliations:** Department of Operative Dentistry and Endodontics, Faculty of Odonto-Stomatology, University of Medicine and Pharmacy at Ho Chi Minh City, Ho Chi Minh City, 700000 Vietnam

**Keywords:** Dental equipment, Dental radiology, Endodontics

## Abstract

The aim of this study is to investigate the accuracies and the agreements of the 3D Endo software, conventional CBCT software Romexis Viewer at three voxel sizes, and the EAL ProPex Pixi in endodontic length measurements. Three hundred and twenty-nine root canals in 120 intact human extracted molars were accessed. The actual lengths (AL) and electronic lengths (EL) were measured using the ruler and electronic apex locator (EAL), respectively. Teeth were scanned using the CBCT at different voxel sizes (0.075, 0.10, and 0.15 mm). Root canal lengths were measured using 3D Endo with proposed length (3D-PL) by software, corrected length (3D-CL), Romexis Viewer. The Fisher’s exact test, paired t-test and Bland–Altman plots were calculated to detect the agreements of the four methods with AL measurements. The ProPex Pixi measurements obtained the highest accuracy in the range of ± 0.5 mm. There was agreement between the 3D-PL and the 3D-CL with AL measurements at voxel size of 0.15 mm and at voxel size of 0.10 mm, respectively. The CBCT Romexis Viewer measurements agreed with AL at three voxel sizes. The conventional CBCT measurements using Romexis Viewer and dedicated software did not reach to the 100% accuracy in the range of ± 0.5 mm.

## Introduction

Endodontic treatment requires not only knowledge of and familiarity with root anatomy, root canal morphology and their alterations, but also the exact lengths of these root canals to ensure the optimally removal of canal components in the root canal preparation phase^1^. An appropriate working length (WL) is important in confining the instrumentation inside the radicular space, restricting extrusion of debris and ensuring proper obturation^1^. The end point of root canal procedure is apical constriction, an inconstantly anatomic landmark. Regardless of this inconsistent feature, an effort should be incessantly performed to enclose root canal treatment inside the canal space. The mission in locating the endpoint of endodontic treatment has usually required the combination of many contemporary modalities and operator skills.

Traditionally periapical (PA) radiograph has been tough in many dental schools for working length determination, besides being utilization as a diagnostic method. However, this has certain shortcomings. This analogue or digital radiograph presents a two-dimensional plenary projection of a three-dimensional structure, leading to misinterpret actual condition, identify root apex incorrectly, overestimate WL, and manage endodontic therapy hardly^1^.

With the coming out of “multiple frequencies” or “ratio method” mechanisms, the following generations of electronic apex locator (EAL) overbalance the early generations, which base on the far simpler mechanisms^2^. Although the reliability and accuracy of the EAL are better than that of traditional radiograph, the performance of the EAL might be falsified by certain situations such as obstruction of canal, or complexities in anatomical features^3^.

Cone-beam computed tomographic (CBCT) scan is a contemporary radiographic imaging system and has been advocated that can overcome shortcomings in conventional radiographic methods^4^. Data available from the pre-existing CBCT scan allows for precise diagnosis, proper treatment planning, and predictable canal instrumentation^4^. CBCT is also an accepted modality to evaluate and visualize the complicated morphology of an individual tooth^5^.

However, reports from several studies in the literature regarding the precision of CBCT measurements compared with that of other modalities reach no concurrence^1,6,7^.

Recently, 3D Endo software (Dentsply Sirona, Johnson City, TN, USA) has been developed for endodontic treatment planning with high complex situations^8–10^. The requirements of minimum resolution of 200 µm and standard DICOM are compatible with the 3D Endo software with an intuitive, lively, attractive interface for analysis.

3D Endo software is introduced and dedicated for complex endodontic cases in the clinical situation. This software, with an intuitive, friendly interface and clear instructions step-by-step throughout procedure, completely satisfies all requirements for all cases, especially in the simulated endodontic education^8^.

WL determination with the canal pathway is one of the most creative characteristics of the 3D Endo software. The function of manual adjustment of length whenever the suggested length is not satisfied is also a valuable feature of the software. This feature is developed in the effort of maximum reduction of operator’s errors in WL determination. Depending on the curvature levels of the canal after adjusting the canal pathway, the operator can estimate proper length of instrumented canal to correct the WL for following steps. The virtual canal pathway displayed on the screen of the computer assists the operator in effective visualization and management of the root canal instrumentation^8,10^.

One of the most important of CBCT data is the voxel size used for scanning. This parameter can affect the image quality and the accuracy of the endodontic length measurement^6,11^. The smaller voxel size is, the higher radiation dose the patient burdens^12^, and whether there is a most appropriate voxel size that balances the accurate level of measurement, the agreement of measurement with the actual length, and the radiation dose. There was limit data on the accuracy of different voxel sizes in endodontic length determination^11^ and not any study on that in association with 3D Endo software. Although 3D Endo software was studied for canal length measurements, that was investigated with the premolar^9^ or with just only one voxel size^13^.

The aim of this study is to investigate the accuracies and the agreements of the 3D Endo software, conventional CBCT software Romexis Viewer at three voxel sizes, and the EAL ProPex Pixi in endodontic length measurements.

## Materials and methods

The present study was approved by the Research Ethics Committee of the University of Medicine and Pharmacy at Ho Chi Minh City, Vietnam. The study was performed in accordance with relevant guidelines and regulations. The study acquired the intact human extracted molars obtained from many hospitals for many reasons, with the informed consents were obtained from all participants. Using the data from the previous study^1^, and the sample size calculation in Bland–Altman plot submenu of the MedCalc Statistical Software version 19 (MedCalc Software, Ostend, Belgium), the appropriate size was 329 root canals. Total of 120 extracted molars was chosen for the present study.

**Figure 1:**
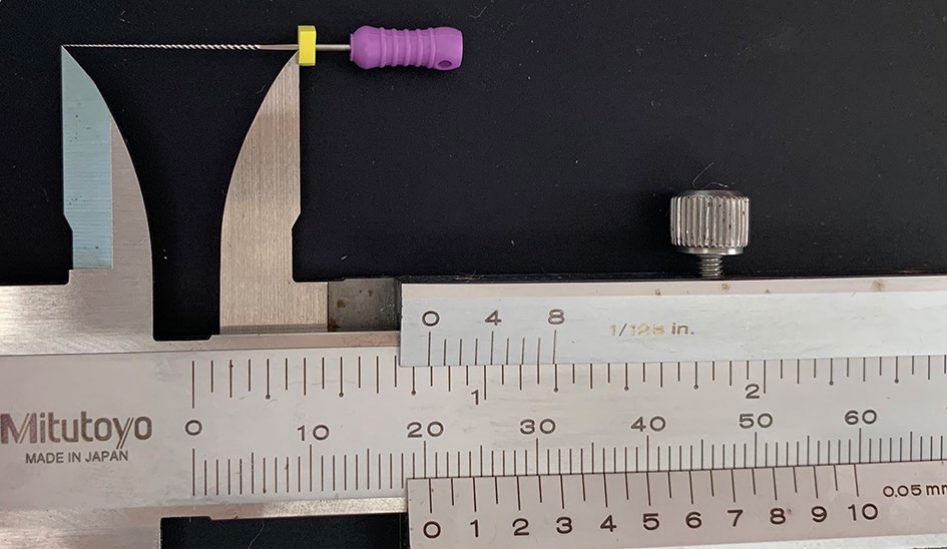
Actual length measurement with the ruler.

Teeth were stored in sterile saline and then cleaned with the ultrasonic scaler Cavitron (Dentsply Sirona, Switzerland), immersed into the 10% formalin solution. Teeth were observed thoroughly under a stereomicroscope to exclude immature apical, cracked, external resorption roots (at a magnification of ten). After being coded with numbers on the crowns, teeth were then ready for cavity access preparation. Cavity access was prepared with the straight-line access concept using the Martin and Endo-Z burs (Dentsply Sirona, Ballaigues, Switzerland). After exposure of all canal orifices was completed, the #10 ISO K-file was introduced into all canals until the tip of the file was visible at the most coronal border of the AF opening under the stereomicroscope (Olympus SZX16, Olympus Corp., Tokyo, Japan). The rubber stop was adjusted to the occlusal reference point, the file was removed from canal, straightened with a forceps, and the length from tip to rubber stop of the file was measured using a digital caliper Mitutoyo (Mitutoyo Corp, Kawasaki, Japan) and recorded as the actual canal length (AL) (Fig. 1). The teeth were then immersed into the freshly mixed alginate tray to prepare for electronic measurements. The root canal lengths were measured using the electronic apex locator ProPex Pixi. The #10 ISO K-file was inserted into the canal until the 0.0-mark lighted up and remained stable for 5 s on the EAL’s screen. The rubber stop was adjusted to the occlusal reference point, the file was withdrawn from the canal and the length was measured as mentioned above. This length was recorded as the electronic length (EL).

Afterwards, all teeth were arranged and immersed in the plastic molds. The 3 mm thick floor of the mold was made by wax and the remaining part was light impression silicone, that reached to the cemento-enamel junctions of teeth. Molds with teeth were then put on the customized shelf and scanned using the cone beam computed tomography (CBCT) (Planmeca ProMax 3D Classic, Planmeca Oy, Helsinki, Finland) with endo mode, 90 kV, 8 mA or 10 mA, field of view (FOV) 50 × 50 mm, at three different voxel sizes of 0.075 mm, 0.10 mm, and 0.15 mm.

**Figure 2:**
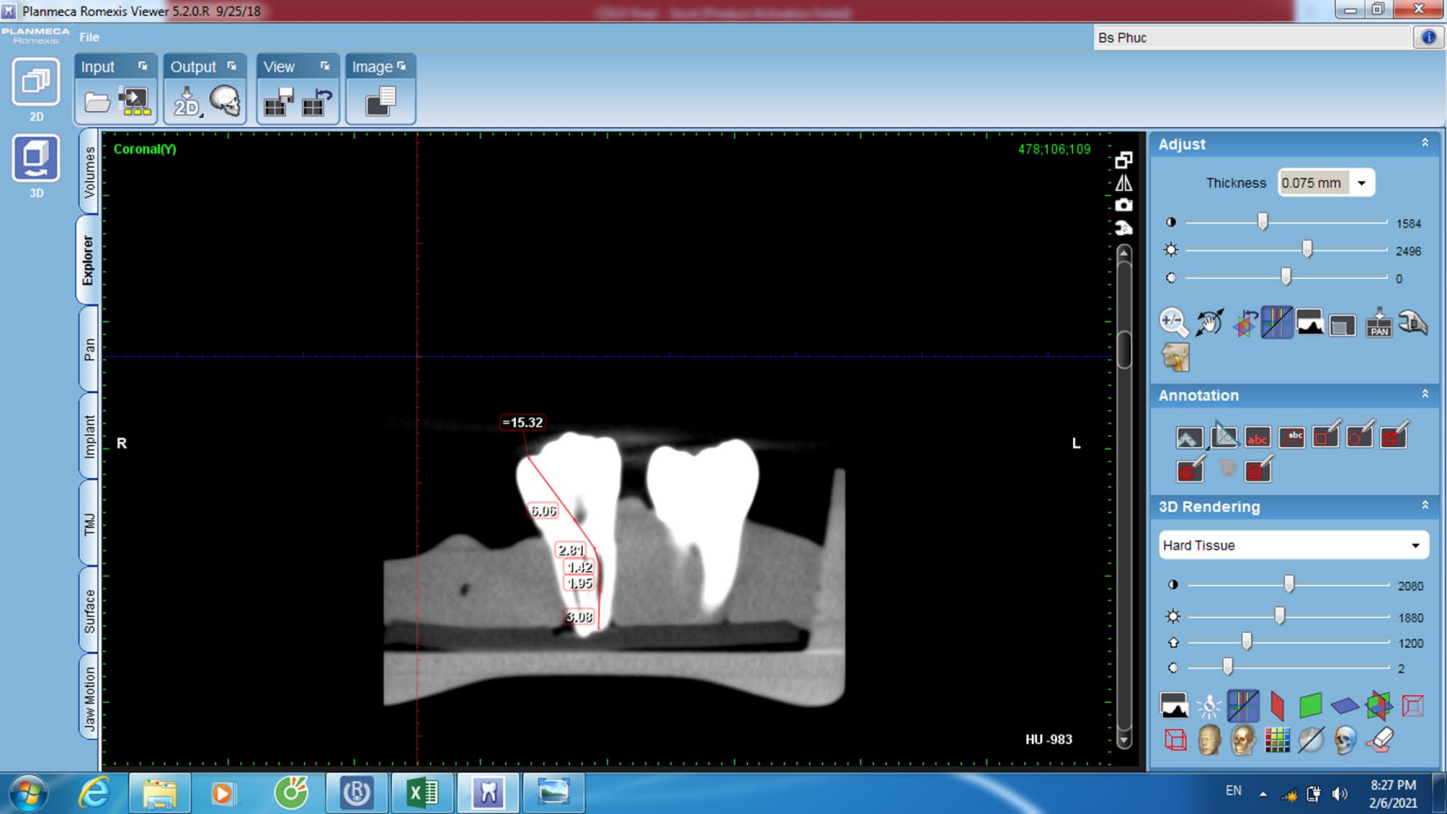
Romexis Viewer measurement on the conventional CBCT software.

In the first section, the CBCT images were observed and analyzed using the Romexis Viewer software from CBCT manufacturer. The slices of the tooth were scanned and observed until the best image of entire length of the canal in bucco-lingual view at the greatest curved angle was selected. The measuring line was drawn from the occlusal reference to the apical foramen (AF), accompanying any deviations from the course of the canal, and was measured in millimeters. Root canal lengths were measured using the tools of Romexis Viewer and recorded as the conventional CBCT length (Fig. 2). The conventional CBCT measurements were performed twice with an interval of two weeks to check the intra-examiner reliability.

**Figure 3:**
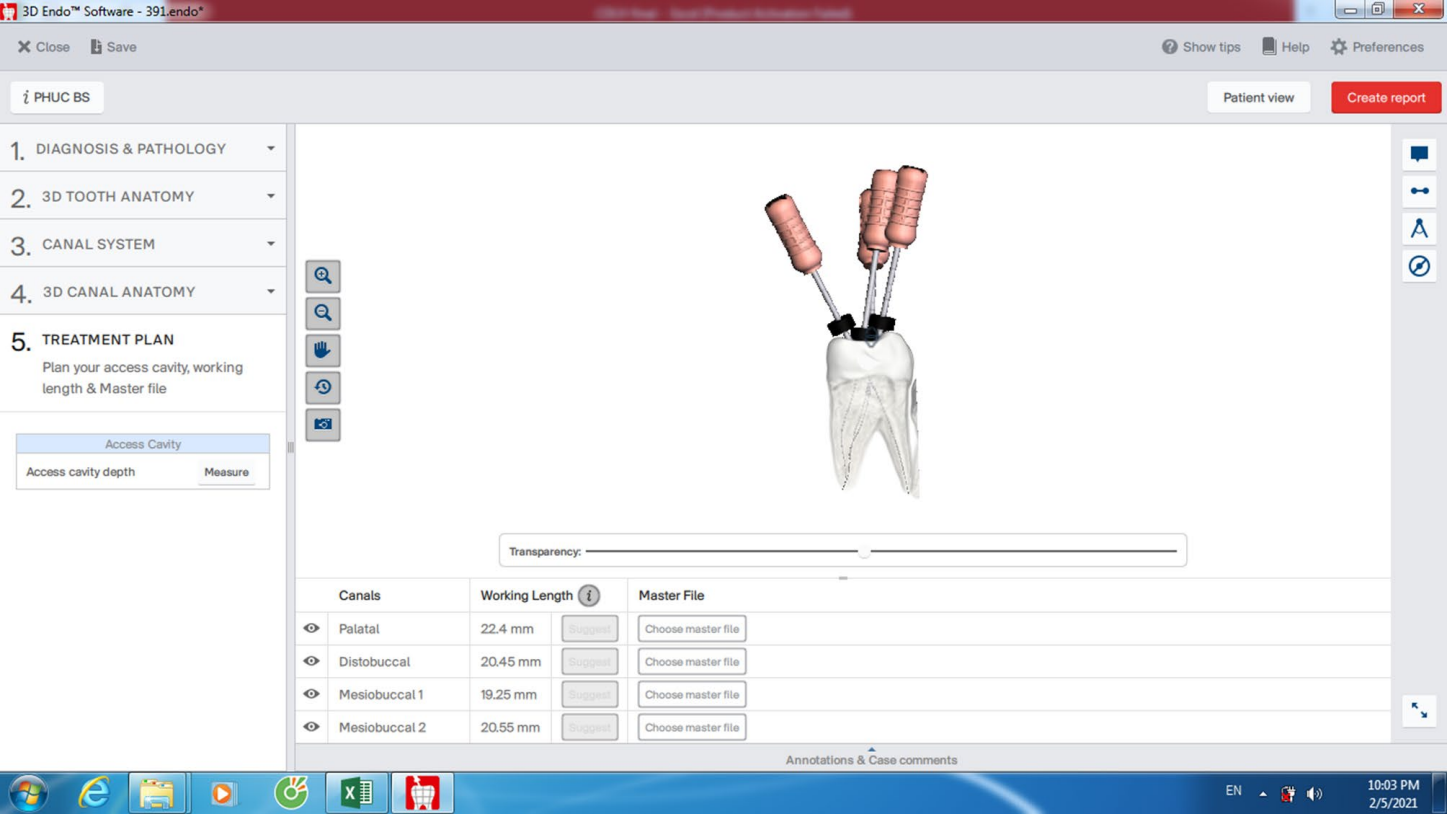
Proposed length measurement on the 3D Endo software.

**Figure 4:**
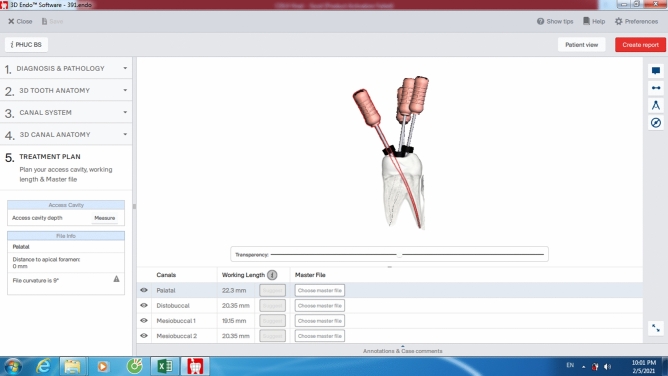
Corrected length measurement on the 3D Endo software.

Afterward, the CBCT data were displayed on the 3D Endo software (Dentsply Sirona, Johnson City, TN, USA) to analyze using its own tools. Tooth’s 3-D image was first isolated with surrounding structure using the proper tools of the software. The operator confirmed the canal orifice and the apex foramen for each canal of each tooth. Once these two landmarks were defined, the automatic line would be drawn immediately by software to connect these two points. The canal pathway was defined by selecting and adjusting the positions of as many points as possible on this line in horizontal and vertical planes from the orifice to apex. This step was performed by operator. The 3D Endo software reconstructed automatically the 3-D image and inside canal system and inserted a virtual K-file reaching to the apex. After adjusting the coronal angulation of the file following the straight-line access concept, the operator pressed the Suggest button to automatically produce the proposed length (3D-PL) (Fig. 3). This length was recorded as 3D-PL. The position of the rubber stop at the proposed length normally was adjusted by the operator for best suitable location, and this length was recorded as corrected length (3D-CL) (Fig. 4). The 3D Endo measurements were performed twice with an interval of two weeks to check the intra-examiner reliability. All Romexis Viewer and 3D Endo CBCT measurements were performed by the same endodontist trained in CBCT diagnostic and 3D Endo applications using the Dell Latitude E7440 system (Dell Technologies, USA).

All measuring data were stored and analyzed using MedCalc Statistical Software version 19 (MedCalc Software, Ostend, Belgium). The data were first screened for normality of distribution using the Shapiro–Wilk test. The intra-examiner reliability was checked using IntraClass Correlation (ICC) index. Fisher’s exact test, Paired t-test, Bland–Altman plots were used for analyzing the data with the Fisher’s exact test for the differences of the percentage of measurements in the range of ± 0.5 mm among groups, Paired t-test for the differences of measurements among experimental groups and the actual length, and the Bland–Altman plots for the agreements of measurements with the actual length.

## Results

The ICC indices for the intra-examiner reliability were greater than 0.96. The proportion (%) of differences between the actual length and the length measurements using CBCTs and the EAL was displayed in Table 1. The highest and lowest proportion of the accuracies in the range of ± 0.5 mm was of the ProPex Pixi and Romexis Viewer (voxel size of 0.15 mm) measurements were 100% and 47.4%, respectively. The Fisher’s exact test showed that there were significant differences among the experimental groups (*P* < 0.05).

**Table 1:** The incidence (%) of differences between the actual length and length measurements using CBCTs and the EAL.

Groups	Voxel size	Shorter than AL N (%)		Equal to AL N (%)	Longer than AL N (%)		± 0.5 mm (%)
		> 0.5 mm	≤ 0.5 mm		≤ 0.5 mm	> 0.5 mm	
3D-PL—AL	0.075	7(2.1)	54(16.4)	24(7.3)	233(70.8)	11(3.3)	94.6^b^
	0.100	7(2.1)	101(30.7)	20(6.1)	169(51.4)	32(9.7)	88.2^a^
	0.150	23(7.0)	119(36.2)	18(5.5)	145(44.1)	24(7.3)	85.7^a^
3D-CL—AL	0.075	4(1.2)	180(54.7)	30(9.1)	113(34.3)	2(0.6)	98.2^c^
	0.100	10(3.0)	149(45.3)	16(4.0)	144(43.8)	10(3.0)	94.0^b^
	0.150	24(7.3)	159(48.3)	10(3.0)	122(37.1)	14(4.3)	88.4^a^
Romexis—AL	0.075	20(6.1)	155(47.1)	2(0.6)	136(41.3)	16(4.9)	89.0^a^
	0.100	58(17.6)	83(25.2)	0(0.0)	126(38.3)	62(18.8)	63.6^e^
	0.150	82(24.9)	68(20.7)	0(0.0)	88(26.7)	91(27.7)	47.4f.
ProPex Pixi—AL		0(0.0)	310(94.2)	10(3.0)	9(2.7)	0(0.0)	100.0^d^

AL, Actual Length; PL, Proposed Length; CL, Corrected Length.

^a,b,c,d,e,f^ Superscript different letters indicated the significant differences at *P* < 0.0. (Fisher’s Exact test).

The proportions of accuracy in the range of ± 0.5 mm reversed to the resolutions of CBCT images for both dedicated and conventional CBCT software, that means, those proportions increased when the voxel size decreased (Table 1).

The 3D-CL measurements reached to highest accurate proportion at the smallest size of voxel when compared to the 3D-PL measurements. Except for the voxel size of 0.15 mm, with the remaining two sizes of voxel, the accurate proportions of 3D-CL measurements were significantly higher than that of 3D-PL measurements (Table 1).

The mean biases, confidence intervals, *P* values in the paired t-test and linear regression analysis, fixed or proportional biases for different methods’ measurements were displayed in the Table 2. Using the analysis method of the previous studies^14–16^, there were five fixed biases and two proportional biases for the present study.

**Table 2:** Mean biases, standard deviations, confidence intervals, *P* values in two statistical tests, fixed or proportional biases for different methods’ measurements.

Groups	Voxel size	Paired t-test			Linear regression	Fixed bias	Proportional bias
			Mean bias	95% CI	P	P	
3D-PL—AL	0.075	0.1082	0.0807 to 0.1357	< 0.0001*	0.0092*	Yes	Yes
	0.10	0.1108	0.0748 to 0.1468	< 0.0001*	0.5449	Yes	No
	0.15	0.0194	− 0.0207 to 0.0595	0.3411	0.9784	No	No
3D-CL—AL	0.075	− 0.0312‏	− 0.0493 to − 0.0130	0.0008*	0.1900	Yes	No
	0.10	− 0.0089‏	− 0.0409 to 0.0230	0.5814	0.4946	No	No
	0.15	− 0.0609‏	− 0.0971 to − 0.0247	0.0010*	0.1202	Yes	No
Romexis—AL	0.075	− 0.0139‏	− 0.0554 to 0.0275	0.5078	0.0324*	No	Yes
	0.10	0.0456	− 0.0135 to 0.1047	0.1299	0.3723	No	No
	0.15	0.0516	− 0.0247 to 0.1279	0.1840	0.8916	No	No
ProPex Pixi—AL		− 0.1695‏	− 0.1770 to − 0.1619	< 0.0001*	0.5573	Yes	No

*Differences at significant level of 0.05.

**Figure 5:**
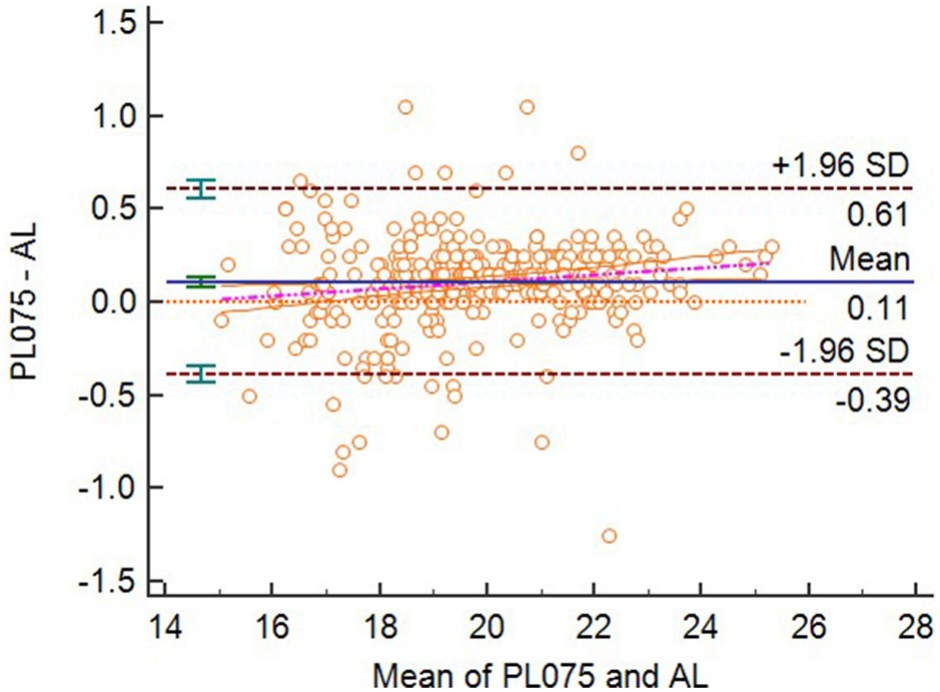
Bland–Altman plot for the agreement of 3D-PL and AL measurements at voxel size of 0.075.

**Figure 6:**
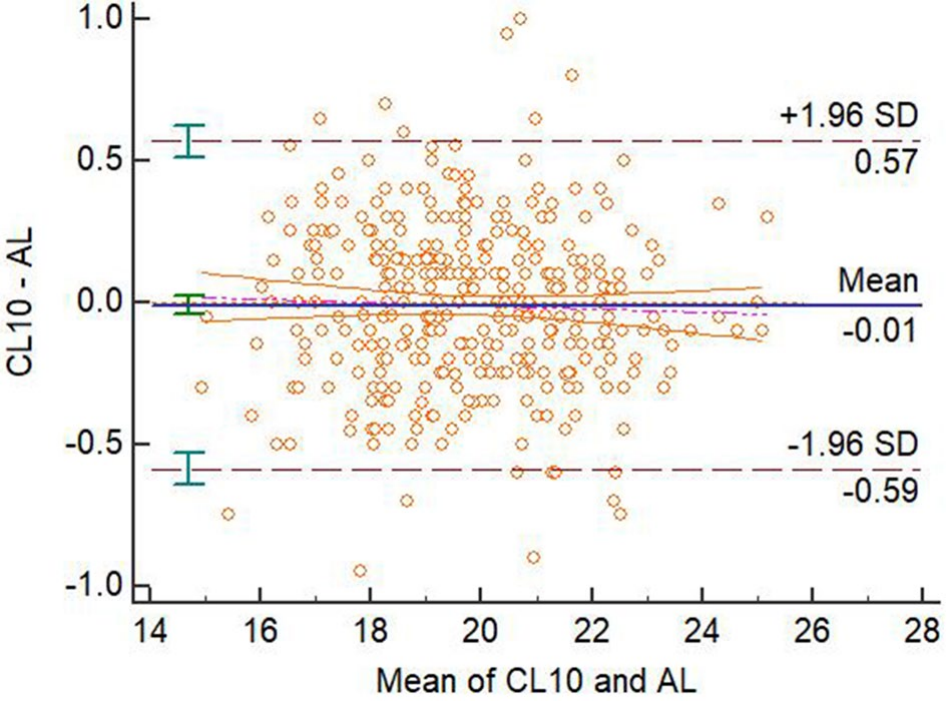
Bland–Altman plot for the agreement of 3D-CL and AL measurements at voxel size of 0.10.

**Figure 7:**
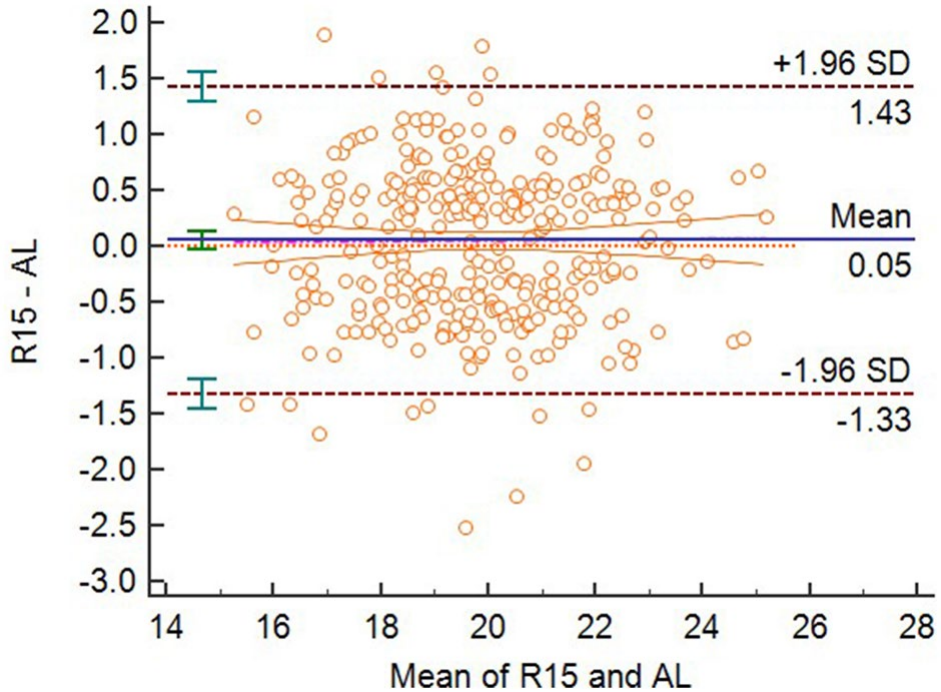
Bland–Altman plot for the agreement of Romexis Viewer and AL measurements at voxel size of 0.15.

**Figure 8:**
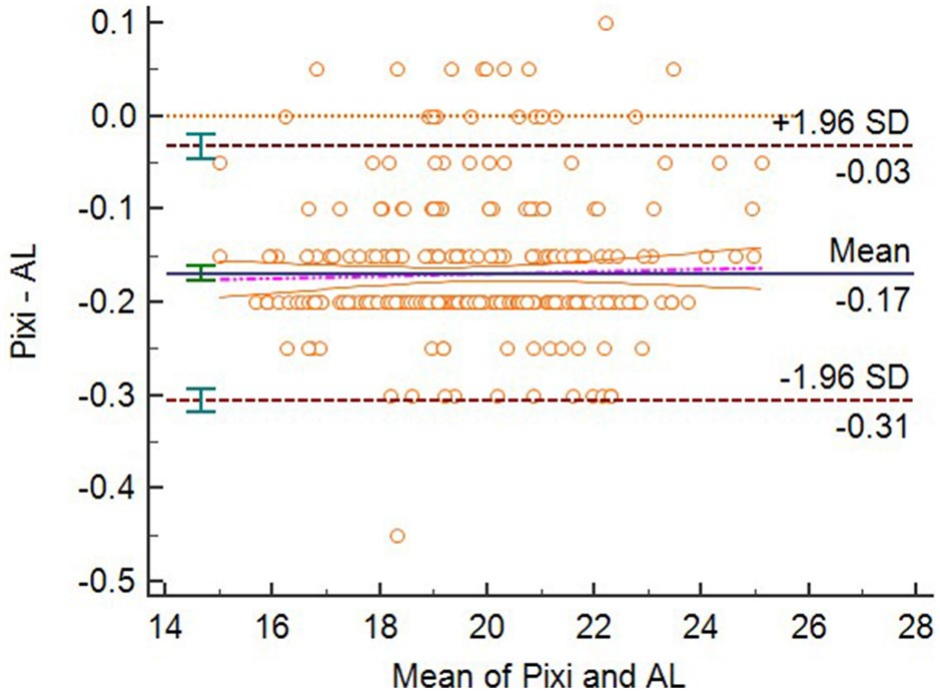
Bland–Altman plot for the agreement of ProPex Pixi and AL measurements.

There were not significant differences in the mean differences between 3D-PL (voxel size of 0.15 mm), 3D-CL (voxel size of 0.10 mm), and Romexis Viewer (all three voxel sizes) and the AL measurements (*P* > 0.05), therefore, these measurements agreed with the AL. All other CBCT and EAL measurements disagreed with the AL. The Bland–Altman plots for the agreements of the four modalities with AL were displayed in the four figures, Figs. 5, 6, 7 and 8.

## Discussion

The results of the present study showed that, in the range of ± 0.5 mm from the apical foramen, the EAL ProPex Pixi was the best accurate modality among the experimental ones. Among the CBCT measurements, the accuracy in the range of ± 0.5 mm was lowest in the Romexis Viewer (the largest voxel size) measurements, using the 3D Endo software.

The important role of the working length determination had never diminished, especially in the existing of more and more modern effective instruments for preparation^17,18^, the complexity of the root canal system^19^. There was fixed bias between the ProPex Pixi and the AL measurements with the means of the EAL measurements were constantly lower than the latter, across the entire range of measurements.

The results of the present study showed that there were the 3D-PL measurements at voxel size of 0.15 mm and the 3D-CL measurements at voxel size of 0.10 mm agreed with the AL. All other 3D Endo measurements, there were fixed biases, like that of the EAL measurements.

The results of the present study also showed that the accuracies of the 3D-CL measurements were higher than that of 3D-PL measurements at voxel sizes of 0.10 and 0.075 mm. The 3D-CL measurements were the WL after correcting the proposed lengths by the operator. The virtual rubber stop on the file was corrected using the anatomic landmarks on the occlusal surface by the operator in the effort of reaching to the most accurate length. The results showed that, at smaller voxel sizes, the adjustments of the rubber stop positions were easier, leading to more accurate correct lengths. This agreed with the results of the previous studies^6,9,11^.

The conventional CBCT Romexis Viewer measurements in the present study obtained the more accurate at smaller voxel sizes, like that of the previous studies^6,11^. The present study used the Bland–Altman plots for analysis of the agreement of the measurements with the AL, differed from the previous studies^6,11^. The 3D Endo measurements also confirmed the similar results in the present study, that the smaller voxel sizes yielded more accurate measurements.

Studies on determination of WL with CBCT commonly used human extracted teeth in dry mandible or in jaw model^6,20,21^. The setting with the dry human mandible is better than other contexts in controlling of some clinical variations such as artifacts caused from position or motion of patient, beam hardening from other surroundings, or noise from other anatomic structures^21^. The arrangement of teeth in the impression tray of the present study does not eliminate completely artifacts from the neighboring teeth in the tray. However, CBCT images are clear and anatomic landmarks are defined easily and exactly owing to of high resolution^11^. The Romexis Viewer measurements agreed with the AL with good mean differences, just higher than that of the best method in the present study. At the present voxel sizes of the CBCT image, the Romexis Viewer measurement was reliable modality for WL determination. This result differed from the other previous studies^1,6,11,21^.

Although the human extracted teeth seem appropriately for evaluation the accuracy of CBCT WL determination, the artificial endodontic training tooth still completely satisfies requirements of this purpose^22^. Authors just select the actual root canal length of the artificial tooth as the gold standard in evaluation of the accuracy of the CBCT WL^22^. The 3D Endo software can improve accurate 3D root canal length determination, however, the operator should check, control, and maintain continuously the working length during the preparation phase to detect changes, especially in severe curved canal^22^.

One important shortcoming with CBCT in endodontic WL determination on the heavily metallic restored tooth is the significant artifact^23^. More artifact means a greater approximate range of length, and in these cases, CBCT provides only an estimate of the length^23^.

Proper knowledge of root canal anatomy and morphology is indispensable for every clinician in endodontics for locating the root canal orifices. CBCT imaging has supplied an exact, noninvasive approach for clinical chairside assessment of root canal anatomy^4^. The 3D Endo software is an effective, quick, and easy modality for identification and visualization of canal trajectories in three dimensions. This software reveals promise in supporting operator for quantifying anatomical complexities preoperatively^10,24^.

Endodontics can be performed at a high level without CBCT imaging, but it cannot be practiced at the highest level^23^. Image-guided endodontics with minimally invasive access and instrumentation is recently recommended by authors for better in preservation as much tooth structure as possible^23^.

The radiation exposure for dental CBCT has been a dramatic reduction as compared to the conventional medical CT. However, this is not the major key to use for the routine examination in endodontics. The benefits from the examination far outweigh the risks related to ionizing radiation exposure should be carefully considered^23^. Although the radiation exposure for each dental examination is less than dose of radiation from other sources, the exposure time is so short, leading to the augmentation of damage. Diagnostic examinations should be performed at the lowest dose of radiation, following the ALARA principle: as low as reasonably achievable^12^. The American Association of Endodontists statement suggests that the risk–benefit ratio is too high for CBCT to be a routine screening tool, even though the radiation levels are low with focused-field device^25^. Therefore, the application of CBCT only for root canal length measurement is not advocated^22^.

The present study used three different voxel sizes for evaluation of the accuracy and agreement of the CBCT measurements compared to the AL. This differed from the previous studies using 3D Endo software^9,22^. One important feature of the present study was the root canals of the human extracted molars with appropriately calculated sample size used for the evaluation, that differed from the other studies^9,11^. However, the present study did not include the dry jaws to simulate the clinical situation and use the intact human molars for a real situation with the pre-existing CBCT data of the patients. Further studies should perform to confirm the effectiveness of the 3D Endo software with better conditions to simulate the clinical situations such as dry jaws, intact human premolars, molars.

The CBCT measurements using 3D Endo with the proposed length and corrected by the software and Romexis Viewer with different voxel sizes did not reach to 100% accuracy in the range of ± 0.5 mm from the actual root canal length.

## Data Availability

The datasets used and/or analyzed during the current study are available from the corresponding author on reasonable request.
